# Thinking like a mountain: A land ethical approach to healthcare resource allocation

**DOI:** 10.1111/bioe.13355

**Published:** 2024-10-06

**Authors:** Alistair Wardrope

**Affiliations:** ^1^ Department of Neurology Sheffield Teaching Hospitals NHS Foundation Trust Sheffield UK; ^2^ Academic Neurology Unit University of Sheffield Sheffield UK

**Keywords:** aldo leopold, Anthropocene, climate ethics, environmental ethics, land ethic, resource allocation, sustainability

## Abstract

Human activity is now having a defining influence on global systems. The Anthropocene epoch requires revisiting our ethical presuppositions to understand our relationship to the earth's life support systems. The Land Ethic of Aldo Leopold proposes an ethic that is diachronic, holistic, and biocentric, in contrast to the synchronic, individualist, and anthropocentric axioms of mainstream bioethics. I argue that these features of the Land Ethic make it more suitable to engage with the ethics of healthcare resource allocation in the Anthropocene; that understanding sustainability in a Land Ethical fashion requires that we view it as placing a side‐constraint on all permissible healthcare resource use such that this use remains within planetary boundaries; and outline how this might re‐shape debates around healthcare resource allocation.

*The cowman who cleans his range of wolves does not realize that he is taking over the wolf's job of trimming the herd to fit the range. He has not learned to think like a mountain. Hence we have dustbowls, and rivers washing the future into the sea*.^1^

*Thinking the Anthropocene […] means abandoning the hope of emerging from a temporary ‘environmental crisis’ […] The irreversible break is behind us, in that brief and exceptional moment of two centuries of industrial growth. The Anthropocene is here. It is our new condition*.^2^

## 1 INTRODUCTION

The majority of the history of human development—including all of written history and development of civilisations—has occurred within a single geological epoch, the Holocene. Within this period, global temperatures have been relatively stable, warmer than the preceding glacial age, with higher sea levels. While humans were found across most of the globe at the start of the Holocene, human activity had little impact on the overall state of the climatic and stratigraphic signals by which the period is defined.^3^

The same can no longer be said. Whether measured in terms of global mean surface temperatures, atmospheric greenhouse gas (GHG) concentrations, widespread deposition of naturally rare radioisotopes from nuclear weapons testing, or extinction rate, the driving perturbations to earth systems are now anthropogenic in origin, resulting in a proposed (though—at time of writing—officially rejected) new geological epoch defined by these human influences; the Anthropocene.^4^ In this context, the dominant ethical tools of the Holocene—in which the natural environment features as little more than inexhaustible resource—offer little guidance, ill‐equipped as they are to handle what Isabelle Stengers has called ‘the intrusion of Gaia’.^5^ Debates around healthcare resource allocation that assume a discrete pool of resources that can be assigned to a set range of problems affecting a countable number of identifiable individuals will struggle with the moral arithmetic of evaluating how our actions affect innumerable other present and future persons in poorly quantifiable ways through their distributed environmental impacts.^6^

One approach to addressing the ‘intrusion of Gaia’ into bioethics is found in a reformist approach—adding principles oriented to sustainability or environmental harm to modify the existing moral calculus.^7^ My objective is instead to outline a different approach—one that sees the ‘irreversible break’^8^ of the Anthropocene as motive to modify the axioms of bioethics, and our perceptions of the moral landscape. Beyond that irreversible break, our environment is more than inexhaustible resource for our needs and infinite sink for our waste; it is intimately entwined with our health and wellbeing, such that our continued flourishing cannot be understood except through our dependence on, and effects on, our ecological community.

Such a view of the ecological community is central to the Land Ethic of Aldo Leopold. While heavily influential in the work of Van Rensselaer Potter (who introduced the term ‘bioethics’ in Anglophone discourse), subsequently Leopold's work has received comparatively little attention in medical ethics. In perhaps the most famous line of his *oeuvre*, Leopold claims:

A thing is right when it tends to preserve the integrity, stability, and beauty of the biotic community. It is wrong when it tends otherwise.^9^

The Land Ethic emphasises ‘stability’—it is a conspicuously *diachronic* ethic, in contrast to the *synchronic* focus on the ‘punctate decision’^10^ that dominates mainstream bioethics. Its primary unit of ethical evaluation is the ‘community’—making it a *holistic* ethic (‘holistic with a vengeance’,^11^ according both to some proponents and detractors), in opposition to the methodological individualism common to dominant approaches. And that community is not solely human, but ‘biotic’—the Land ethic is *biocentric*, against the anthropocentric norm.

In this study, I offer an outline of a Land Ethical approach to distribution of healthcare resources. I do not do so because this is the most important or overriding consideration for incorporating environmental ethics into biomedical ethics—as I hope is clear below, the Land Ethic identifies the view of environment as simply a resource that is instrumentally valuable to human health interests as one of the crucial problems contemporary bioethics faces. Nor do I wish to suggest that health workers need confine themselves only to such familiar questions of distributive justice in health care to understand their responsibilities in the Anthropocene condition—I argue elsewhere that the view I present dissolves boundaries between ‘personal’ and ‘professional’ responsibilities and make advocacy for planetary health as much health workers' concern as the traditional questions of biomedical ethics.^12^ Instead, I intend to demonstrate how even such familiar questions must be reframed, highlighting the ecological context of healthcare practice.

I argue (Section 2) that the ethical challenges of healthcare resource allocation in the Anthropocene require a diachronic, holistic, and biocentric ethic such as the Land Ethic. I then show (Section 3) that empirical evidence of anthropogenic influences on earth's life support systems, and the planetary boundaries^13^ these impose, require that an ethical understanding of sustainability worthy of the name requires that it function as a side‐constraint to permissible courses of action—defining a ‘safe operating space’^14^ for all medical and ethical deliberation—rather than being a principle that can be weighed against (and potentially out‐competed by) other considerations. Finally (Section 4), I give examples of how a Land Ethic in practice could shift our approach to resource allocation in health care, both in evaluation of the goals of health care and the understanding of health care's role in wider society. From this perspective, the challenge for the ethics of healthcare resource allocation then becomes twofold: how to achieve moral consensus on which holistic outcomes are most appropriately valued, within the determined resource envelope; and how healthcare needs to respond to the 95%+ of resources not directly consumed by healthcare, but that contribute to shifting ecosystems beyond safe planetary boundaries that preserve the ‘integrity, stability, and beauty of the biotic community’.

## 2 A LAND ETHIC FOR HEALTHCARE RESOURCE ALLOCATION

The Anthropocene poses a profound challenge to ethical theorising, what Stephen Gardiner has called a ‘perfect moral storm’.^15^ While not seeking to engage in detailed exegesis of the Land Ethic,^16^ in this section, I will outline some features driving the moral storm in the ethics of healthcare resource allocation and highlight how the Land Ethic may be well positioned to respond to these. I argue elsewhere that mainstream bioethical theories such as principlism are overwhelmingly individualistic, anthropocentric, and individualistic in their application^17^; here I argue that an ethic of resource allocation in the Anthropocene must challenge these features.^18^ I do not here intend to persuade the sceptic that they must adopt the Land Ethic (I have argued in its favour elsewhere),^19^ but highlight that it has attractive features for addressing the challenges of sustainable resource allocation that are notably absent in mainstream bioethics.

### 2.1 Diachronic ethics

In their totemic *Principles of Biomedical Ethics*, Beauchamp and Childress take the archetypal structure of moral questions in bioethics to be that of the ‘moral dilemma’—a single decision in which competing moral considerations weigh in favour of different options amongst a small number of possible decisions, affecting a clearly identifiable—and usually small—number of parties.

Feminist critics of mainstream bioethics have long highlighted that this *synchronic* bias—focusing on isolated decisions involving only parties present at a single time point—neglects more temporally extended questions of moral relevance. Virginia Warren characterises the emphasis on dilemmas as a focus on ‘crisis issues’, neglecting the temporally extended ‘housekeeping issues’ that shape how dilemmas arise and who is involved in them.^20^ Quill Kukla (writing as Rebecca Kukla) meanwhile points out that the focus on these ‘punctate decisions’ neglects the fact that such decisions exist ‘within a larger pattern of normative relations between the patient and her health practice’.^21^ Furthermore, choices made at these punctate decisions go on to re‐shape those relations and that decision‐making context.

A diachronic ethic is a *sine qua non* of bioethics fit for the Anthropocene; the Brundtland Commission definition of sustainability is ‘meeting the needs of the present without compromising the ability of future generations to meet their own needs’.^22^ However, existing ethical tools often struggle to evaluate such temporally dispersed claims. The classical problems of distributive justice in healthcare concern (for example) which person is the beneficiary of an organ transplant or intensive care bed—but they do not consider how the resource usage in providing such care has degraded water supplies elsewhere, consumed finite supplies of rare earth minerals, or contributed to patterns of high energy consumption that drive climate change.^23^

The Land Ethic is conspicuously diachronic. It identifies that humans, like all living things, depend upon resilient ecological networks (the ‘land’ of the Land Ethic):

Land… is not merely soil; it is a fountain of energy flowing through a circuit of soils, plants, and animals. Food chains are the living channels which conduct energy upward; death and decay return it to the soil. The circuit is not closed; some energy is dissipated in decay, some is added by absorption, some is stored in soils, peats, and forests, but it is a sustained circuit, like a slowly augmented revolving fund of life.^24^

The disruption of these circuits threatens the flourishing of all its components; therefore, Leopold argues, the temporally extended integrity of the circuits themselves must be valued.

It is not just a failure of the moral apparatus that results in this situation, but a failure of the moral imagination. Peter Singer famously identified how easily proximity and immediacy can manipulate our moral motivations (how much more compelling is the need to rescue the child drowning in a pond in front of us, than the one starving in a famine on the other side of the globe) and argued for the normative irrelevance of these factors.^25^ How much more difficult, then, to feel the obligation to act on behalf of putative, ill‐defined future others, expected to suffer the consequences of unabated environmental crises?

It is for this reason that Leopold, in one of his most famous essays, calls for us to ‘think like a mountain’—to broaden the spatial and temporal scale of our moral lens, such that we do not ‘wash the future into the sea’. As with much of Leopold's writing, the implications of this exhortation are not spelled out in detail; rather, it serves as invitation to refigure our perspective of the moral landscape. Leopold recognised that our moral motives require affective or conscientious engagement with the subject of our moral responsibilities to be persuasive:

Obligations have no meaning without conscience, and the problem we face is the extension of the social conscience from people to land.^26^

To ‘think like a mountain’ is an invitation to extend our moral sentiments to those at a temporal and spatial distance from us, by seeing not ‘putative, ill‐defined future others’, but rather the wellbeing of a single, temporally persisting entity within which we are closely enmeshed—the community that surrounds and sustains us. It is also to see that this community is more than human and has value in, and of, itself—I explore the implications of these more in the next two sections.

### 2.2 Biocentrism

Leopold's invitation to extend our consciences from people to land asks us to see ourselves as part not just of a human community, but a ‘biotic’ one—comprising humans, but also all living and physical systems with which we interact and upon whose wellbeing we depend. Humans are not ‘conquerors’, but ‘plain member and citizen’ of that community. This makes the Land Ethic biocentric—non‐human life has intrinsic, non‐instrumental moral value.

One can make both anthropocentric and biocentric arguments for such a biocentrism. Anthropocentrically, failure to acknowledge the importance of the non‐human elements of our community is ultimately self‐defeating:

[We] have learned (I hope) that the conqueror role is self‐defeating … it is implicit in such a role that the conqueror knows, ex cathedra, just what makes the community clock tick, and just what and who is valuable, and what and who is worthless, in community life. It always turns out that he knows neither, and this is why his conquests eventually defeat themselves.^27^

The considerations that motivate most contributions to this special issue—and that are explored further below—are sufficient evidence of this. It is precisely the assumption that humanity is the conqueror and sole manager of our ecosystems that leaves us confronting the perfect moral storm of the Anthropocene.

Less instrumental justification comes from the acknowledgement that we do not exist outside of the non‐human biotic community, but are essentially interdependent. This is not merely in the sense of depending on ecosystems as ‘life support systems’. Jonathan Beever and Natalie Morar argue that the ‘individual’ as conceived in mainstream bioethics is *constitutively* ecological, pointing to such influences as the microbiome on desire, preference, and value to demonstrate that the crucial aspects of the autonomous self presupposed by that ethic are inseparable from ecosystemic context.^28^

Ultimately, however, determinations of intrinsic value are settled not by argument, but by how we see the world. Beauchamp and Childress feel no obligation to argue for the positive value of individual human lives, considering that to be accepted by all ‘morally serious persons’.^29^ As such, Leopold saw the Land Ethic to require as much moral education as moral argumentation—to help people to see the more‐than‐human world as bearing intrinsic value. To that end, the Land Ethic emphasises direct engagement with our biotic communities and an appreciation of their beauty—to ‘build receptivity into the still unlovely human mind’.^30^

Whether or not we adopt a biocentric ethic of healthcare resource allocation, therefore, is as much a reflection of how we see humanity situated in the world—as ‘conqueror’ or ‘plain member and citizen’ of our biotic communities. However, in the context of present global environmental crises, the hardened anthropocentrist would do well to heed Leopold's warning that the ‘conqueror role is self‐defeating’.^31^ Accepting a place for biocentrism places the challenges of sustainability to the forefront—the ecosystemic implications of our resource use, rather than being ‘externalities’ that can be factored into a greater or lesser extent into our moral reasoning, become core components of our moral calculus.

### 2.3 Holism^32^

As referenced above, ‘thinking like a mountain’ does not simply mean thinking diachronically—it also involves treating the community as a whole as having a value not merely reducible to that of its individual members. This feature of the Land Ethic is attractive insofar as it helps to circumnavigate some notorious evaluative challenges that confront the evaluation of temporally extended distributive justice considerations. These include the discounting rate (how much less value does a person enjoy from given resource use in the future, *simply because they are in the future*)^33^; the mere addition paradox (can utility be maximised by creating more individuals, even if each is individually better off); and the non‐identity problem (if our acts affect which future individuals come into being at all, how do we evaluate in which conditions they are better or worse off—if non‐identical individuals exist in different scenarios?)^34^ A diachronic ethic for resource allocation must offer some means for negotiating such challenges.

These problems arise, however, in large part because they assume that claims of distributive justice must be evaluated on an *individual basis*. The non‐identity problem arises because we cannot readily evaluate the welfare of individuals across different future scenarios who do not exist in all scenarios; the mere addition paradox because overall welfare is a matter of ‘mere addition’ of that of individuals. To establish how *we* are doing, one looks at how *you* are doing, and how *I* am doing, and combines these. This ‘methodological individualism’—what J Baird Callicott calls the ‘Smith and Jones paradigm of ethics'^35^—is so prevalent in bioethics as to have become almost axiomatic.^36^ Even many of those (such as David Resnik and Cristina Richie) who explicitly attempt to reimagine bioethics to incorporate ecosystemic and non‐human concerns frame their discussions of justice and value ultimately in terms of individuals.^37^ Yet it is not the only way of evaluating outcomes.

As per the passage quoted in the introduction, the basic unit of moral evaluation according to the Land Ethic is not the individual, it is the ‘biotic community’. Leopold highlights that the very idea of the abstract human individual, whose welfare can be determined independent of their environment, is ecologically illiterate:

Ecology tells us that no animal—not even man—can be regarded as independent of his environment. Plants, animals, men and soil are a community of interdependent parts, an organism … Mr Babbitt is no more a separate entity than is his left arm, or a single cell of his biceps.^38^

Evaluating outcomes *holistically* in this fashion—not as the aggregate of outcomes for individuals, but as an entire system—sidesteps the challenges of these problems for individualistic ethics. The non‐identity problem arises only when we focus only on individual humans—but the Land Ethic exhorts us to focus instead on the *same* ecosystem and promote its stability and flourishing; while that system may contain different individuals according to different courses of action, the referent of the whole system remains the same.^39^ Holistic evaluation respects and explains the intuition that there is something ‘repugnant’ with the conclusion of the mere addition paradox. Any ecosystem has a finite carrying capacity for individuals occupying a given ecological niche, and indefinite multiplication of individuals beyond this capacity will destabilise the overall system, just as does the ‘cowman who cleans his range of wolves’ in the quotation with which this article opens.

A holistic ethic is therefore an attractive one for the ethics of resource allocation, insofar as it dissolves some of the paradoxes of moral mathematics that arise from solely individualistic ethical evaluation. The question remains, however, of what level comprises the relevant ‘whole’ for the holism of the Land Ethic.^40^ Much of Leopold's discussions of the land communities to which he wishes to extend moral concern are essentially local or regional—an individual river, or the farmland of a particular North American county. For Callicott (*inter alia*), this localism is both essential to the Land Ethic, and a barrier to its engagement with global environmental health threats. Callicott suggests that the kind of affective engagement with the environment necessary to motivate a biocentric ethic required above functions only at the local scale, and—while he considers adopting a holism that views the Earth itself, a ‘proto‐Gaia’, as the relevant whole—he ultimately rejects this as ‘a leap beyond both the spatial and temporal limits of ethics and the spatial and temporal scales of anthropogenic global climate change’.^41^ The entire planetary ecosystem is too abstract and removed from our daily experience to identify with as a subject of moral concern.

This response, however, asserts too parochial a view of the land community and too abstract a conception of Leopold's ‘proto‐Gaia’. Central to his ‘biotic view of land’ is the relationships of interdependence that tie different organisms within the land community together in ‘a fountain of energy flowing through a circuit of soils, plants, and animals’.^42^ These interdependences define the whole of the community—and they also serve to extend our moral concern beyond the parochial. Local ecosystems are not closed—whether through the flow of rivers from source to sea, the spread of seed by transiting animals or the flight of migratory birds from pole to pole—the local is part of the global, and it is by seeing the value of the local that we appreciate that of the global. In Leopoldian terms:

Every March since the Pleistocene, the geese have honked unity from […] Sacramento to Yukon […] By this international commerce of geese, the waste corn of Illinois is carried through the clouds of the Arctic tundras, there to combine with the waste sunlight of a nightless June to grow goslings for all the land between. And in this annual barter of food for light […] the whole continent receives as net profit a wild poem dropped from the murky skies upon the muds of March.^43^

By entering into a direct, caring relationship with the geese who pass through his farm in the Spring, Leopold's sphere of moral concern is spread from Sacramento to Yukon. Far from preventing moral engagement with the global, an appreciation of the intensely local gains entry to the global community. In the next section, I will attempt to outline a means of operationalising this global holism.

## 3 SUSTAINABILITY: PRINCIPLE OR PREREQUISITE?

The Land Ethic therefore proposes an approach to healthcare resource allocation that is diachronic, holistic, and biocentric. In this section, I will discuss its implications for framing sustainability considerations.

Various authors have attempted to construct a ‘sustainability’ principle or set of principles suitable for bioethical application.^44^ Broadly speaking, they treat the environment as a resource pool to be drawn upon for human needs and mandate that the resource pool not be depleted in meeting present needs such that it jeopardise the ability of future generations to meet theirs. Munthe, Fumagalli, and Malmqvist outline a ‘generic sustainability principle’ (GSP)—a formalism they propose any positive, substantive principle of sustainability in bioethics should be able to adhere to:

GSP: [I]f a resource allocation pattern at time t_1_ produces negative dynamic effects at time t_2_, this to some extent counts against this pattern at t_1_, and in favour of resource allocation patterns at t_1_ with no or weaker negative dynamic effects at t_2_.^45^

In the context of the GSP, ‘dynamic effects’ are those actions at an earlier time that increase or decrease the total resource pool at a later time. They go on to elaborate multiple ways in which such a principle could be made substantive: (i) as side‐constraint, ruling out excessively unsustainable actions with lexical priority over other principles; (ii) by gradual weight, being entered into moral deliberations alongside, and potentially in competition with, other ethical principles; (iii) through ‘rational savings’, acting ‘on a metalevel’ by mandating artificial reduction of the resource pool at *t*_1_, to compensate for predicted negative dynamics affecting *t*_2_; or (iv) through ‘insurance’, insisting that resource allocation protocols also pay into an insurance scheme that protects against future damages at *t*_2_.

While the GSP seems sufficiently abstract as to encompass all attempts to flesh out a bioethical sustainability principle, in fact its structure ignores perhaps what is most morally compelling for sustainability. Briefly, the GSP assumes that the resource allocation decision problem at *t*_2_ remains *relevantly similar enough* to that at *t*_1_ in order to be able to establish the relative sizes of resource pools and healthcare needs—in the metaphor Munthe et al. employ that we can compare the resource ‘pies’ and their ‘slicing’ across time. This is clear in both their positive (e.g., vaccination programmes reducing future acute healthcare need) and negative (e.g., antibiotic overuse driving resistance, increasing future health need) examples of dynamic effects in resource allocation. In at least two of the elaborations (iii) and (iv), there is also an assumption of *commensurability of benefits and harms*—that resources set aside, or invested in insurance, at *t*_1_, can adequately compensate for any negative effects of healthcare activity experienced at *t*_2_.

The first assumption is that, while what we do now may create better or worse worlds in the future, they will be basically the same kind of world. It rejects the possibility of *transformative* actions—decisions (e.g., resource allocations) at *t*_1_ such that the ‘needs’ and ‘resources’ at *t*_2_ are so utterly different from *t*_1_'s that comparison is meaningless.

Transformative actions may be unfamiliar to policy makers, thinking on timescales of fiscal years; but they are familiar to conservationists, or one thinking like a mountain. In Leopold's work, he provides repeated examples of how neglecting the possibility of transformative action can lead to environmental catastrophe: it is one thing to calculate how clearing forest and predator control to boost grazing herd sizes or game stocks now may affect future stock yields; but another to see the entire landscape turned to a dustbowl, or ‘rivers washing the future into the sea’.^46^ Likewise, conservation shows the falsehood of the commensurability of values—when one dams a river to generate fertile farmland, or clears boreal forest to extract fossil fuels from the oil sands underneath, one is not trading off two fungible goods; one is destroying unique and irreplaceable ecosystems in exchange for food or fuel.^47^

Considering the dynamics of healthcare's effects on planetary systems makes it clear that resource allocation can be far more transformational, and incommensurable, than the GSP would allow. By definition, the Anthropocene is the period in which human activity has a defining influence on the paths of earth systems. These biological, chemical, and physical systems overall determine the stability of the earth system as a whole. A leading framework for modelling these earth systems defines this stability in terms of nine ‘planetary boundaries’—climate change, biosphere integrity (or biodiversity), land system change, freshwater change, biogeochemical (chiefly nitrogen and phosphorus) flows, ocean acidification, atmospheric aerosol loading, stratospheric ozone depletion, and the generation of novel entities (the environmental presence of compounds—for example, microplastics or organic pollutants—that would not exist without human activity).^48^

Of these planetary boundaries, some of them (e.g., biosphere integrity,^49^ atmospheric aerosol loading,^50^ and climate change)^51^ are of particular concern to health workers for their disproportionate impacts on human health; others (such as the generation of novel entities in pharmaceutical manufacture and nuclear medicine) are disproportionately driven by healthcare resource use. The healthcare implications of each planetary boundary merits detailed ethical exploration in its own right. However, for the purposes of this study, what is important to note is that: they are interconnected, such that perturbations of one system may destabilise others with enduring negative feedback^52^ (e.g., biospheric integrity loss and climate change mutually exacerbate one another)^53^ and that *they are distinct, incommensurable boundaries*. It is not the case, for example, that an increase in greenhouse gas (GHG) emissions at time *t*_1_ can be ‘offset’ by measures that may improve biospheric integrity at *t*_2_; rather, each boundary represents a necessary condition for continued thriving of earth systems. In the terms of the team who originally defined the framework, the planetary boundaries represent a ‘safe operating space’ for humanity. Outside this safe operating space, negative feedbacks and interactional effects between boundaries risk irreversibly destabilising systems, much as Leopold's plains turned to dustbowls.

Viewed from the perspective of the planetary boundaries framework, the GSP is inadequate to capture what is morally important about sustainability. Returning to the Brundtland Commission's definition, sustainability should—if nothing else—avoid compromising the ability of future generations to meet their own needs. The planetary boundaries framework describes how those generations need, as a bare minimum, the continued functioning of the earth's ‘life support systems’. Destabilising these would produce catastrophic environmental change to a degree that would be transformative, and likely irreversibly cause incommensurable harms.

This leads us to the conclusion that sustainability cannot be simply another principle to be weighed and traded against other considerations in bioethics' moral calculus; it is a prerequisite for meaningful moral deliberation that takes diachronic considerations seriously. Sustainability in bioethics should function as a side constraint (Munthe et al's option (i)), with thresholds set at the level such that patterns of resource use are consistent with maintaining earth systems within planetary boundaries. Which is to say:

A thing is right when it tends to preserve the integrity, stability, and beauty of the biotic community. It is wrong when it tends otherwise.^54^

In summary, if our bioethics is to incorporate environmental sustainability meaningfully, it must do so by valuing the maintenance of the Earth's ecological systems within planetary boundaries. Beyond these boundaries, potentially transformative changes or incommensurable harms may result that make the kinds of temporal ‘trade‐off's suggested in the GSP unintelligible. By holding the ‘biotic community’ (earth's ecosystems) intrinsically valuable—and considering that community a persisting entity that outlives its individual members—the Land Ethic has such an understanding of sustainability embedded within it. In order to ensure the integrity, stability, and beauty of the biotic community, sustainability must serve as a prerequisite or side‐constraint on other permissible courses or patterns of action.

## 4 HEALTHCARE RESOURCE ALLOCATION WITHIN PLANETARY BOUNDARIES

Thus far, I have argued that an adequate ethic of environmental sustainability for bioethics, in general, and resource allocation, in particular, must function as a side constraint to permissible patterns of resource utilisation; that these side constraints can be defined operationally in terms of the planetary boundaries that describe a safe operating space for Earth ecosystems and that the Land Ethic provides moral justification for asserting the moral value of these boundaries. To conclude, I would like to outline how we might approach debates about healthcare resource allocation in a fashion that could ‘preserve the integrity, stability, and beauty of the biotic community’.

First, it is important to establish that healthcare resource use does indeed have a significant bearing on those planetary boundaries. For this, the evidence is abundant. Globally, for systems for which there is reliable evidence, healthcare accounts for between 1% and 5% of total anthropogenic drivers perturbing systems beyond planetary boundaries^55^; unsurprisingly, these contributions are highly unevenly distributed, for example, the United States alone accounts for 25% of total global health sector GHG emissions (and as such, U.S. healthcare alone produces over 1% of all anthropogenic GHG emissions).^56^

Once this is established, then the question arises of how health needs can best be met, within the constraints of the continued flourishing of earth systems. Two considerations soon become apparent: first, that permissible healthcare resource allocation will depend significantly on changes in other sectors (e.g., the ecological footprint of healthcare differs widely depending on different nations' energy and transport infrastructure)^57^; and second, that the constraints imposed by sustainability are demanding indeed.

Regarding the second consideration, the latest report of the UN Intergovernmental Panel on Climate Change (IPCC) observes that, for >50% chance of limiting surface warming to <1.5C by the end of the century (a level which, while still causing appreciable climatic perturbations and threatening many unique ecosystems, e.g., small island habitats, represents a planetary boundary for climate change) will require drastic decarbonisation by 2030 and net zero emissions by the 2050s.^58^ This is in fact an understatement of the challenge, since—given the disproportionate current and historical contribution of more‐industrialised nations to total environmental degradation—more‐industrialised healthcare systems (with greater ecological footprints) will need to decarbonise even more rapidly given their ‘climate debt’.^59^

If the pool of available resources for health care is thus constrained, one response is then to say that some need will go unaddressed. Cristina Richie, for example, relies heavily on drawing a distinction amongst the present objectives of healthcare provision between ‘needs’ and ‘wants’ and proposes that sustainability requires healthcare delivering the former, but not the latter.^60^ Another response—perhaps more in line with the cultivation of the ‘ecological conscience’ (the kind of moral perception Leopold held central to the Land Ethic that shifts our view of the environment from ‘a commodity belonging to us’ to ‘a community to which we belong’)^61^—is to rethink how those needs are defined. What do humans—*qua* members and citizens of the land community—need from healthcare? Incorporation of the ecological conscience into our ethical deliberations can promote three evaluative changes supporting environmental sustainability of healthcare: a *clarifying* role, highlighting where practices do not align with the values we already hold (due, e.g., to institutional pressures or social norms constraining practice); an *ameliorative* role, highlighting where our (intuitive, or socially‐conditioned) values do not already align with land health, and making a case for their alteration; and an *interpretive* role, inviting us to question our understanding of the relationship between health and health care, and how that compromises healthcare sustainability.

The clarifying role might identify some ‘easy’ wins, finding perceived needs that in fact do not align with our ideas of flourishing individual human lives, let alone biotic communities. For instance, in highly industrialised healthcare systems, it is presently the norm that healthcare expenditures are high in the last year of life, increasing with proximity to death; over 60% of these resources are used in acute, inpatient care.^62^ Cancer patients see ‘exponential’ increases in service use towards death; up to 38% of patients receive chemotherapy or life‐prolonging treatments in the last month of life, while up to 66% do not receive palliative services.^63^ However, when asked explicitly what care people would like to receive as they move towards death, few endorse this model for themselves; pro‐active, early discussions of goals of care—what people want to get out their health care in the later stages of life—appears to reduce high‐intensity interventions (such as intensive care admission).^64^ Likewise, a huge resource burden (over 20% of the carbon footprint of the U.K.'s National Health Service) arises from the use of pharmaceuticals; but many—especially older, multimorbid—patients are unclear what they are taking, why, and whether doing so actually aligns with what they value most.^65^ Other examples of low‐value or wasteful health care have served as focus for many ‘sustainable healthcare’ initiatives.^66^ This role is clarifying not because mainstream bioethics might disagree with these proposals, but because the institutional structures and approaches to decision‐making that operationalise such ethics fail to achieve what we value. While the principlist might in theory endorse reducing low‐value care at the end of life, principlism supports structuring our healthcare institutions in ways that prioritise choice (over value), isolated decisions (over the temporally extended trajectory of a person's life), and technical, resource‐intensive solutions to healthcare challenges.^67^

Beyond this, the ameliorative role identifies patterns of healthcare resource use that reflect a vision of health that does not align with land health. Such considerations are the focus of much of Van Rensselaer Potter's work, particularly in his (heavily Land Ethic‐influenced) *Global Bioethics*.^68^ While it may be possible to address some health problems with technological medical interventions, doing so may in fact reflect a vision of health need that is antithetical to continued ecosystem flourishing. For example, instead of assuming that the real suffering of fertility can be addressed only with in vitro fertilisation or other medical treatments, creating broader social support for other ways of kinmaking (e.g., adoption, or collective child‐rearing) could address such needs in lower‐intensity fashion (indeed, in a way that meets an unmet present need—of those lacking sufficient care now) while reducing future need, by avoiding creation of future consumers. The principlist position's conservative bent—and its enshrining of (a thin, procedural conception of) autonomy—does not permit it to challenge socially dominant norms in this fashion, even if they could better promote human and ecological flourishing.

Lastly, the interpretive role of the Land Ethic could explore whether all health needs are health*care* needs. As persistent debates around medicalisation of different phenomena make clear, nature does not draw a sharp line in the sand between medical problems and other human problems^69^; it is an open question whether addressing any given human need through a biomedical lens is the most effective response, and what social pressures function to position a problem as under the aegis of medicine.^70^ A sustainable ethic of healthcare resource allocation need also address the social, political, and economic pressures—whether they be perverse financial incentives^71^ or attempts at social control,^72^ while health workers need the epistemic humility to acknowledge that the medical view of problems does not address all, or even what is most important, about meeting those problems.^73^

Returning to the first consideration—that healthcare resource use is not determined solely by patterns of healthcare resource allocation—raises a second challenge for sustainability ethics in health care. To practise healthcare within planetary boundaries requires that we change not just health systems, but (*inter alia*) energy, food, transport, and housing systems. That 95% or more of the activities driving us beyond Earth's safe operating space do not arise directly from health care might be taken as cause to abandon the project of sustainable healthcare; if the climate and biodiversity crises are overdetermined, then health systems (like individuals) might feel inclined to say ‘it's not *my* fault’.^74^ However, this is to neglect that patterns of healthcare resource consumption do not just affect how health care is provided; they also shape the systems providing those resources in wider society. Particularly in more‐industrialised nations, healthcare represents a significant component of overall economic activity (up to 17.8% of GDP in the United States).^75^ In many nations (e.g., the NHS in the United Kingdom), national health systems are able to contract services at a national level, at which they are the overwhelming majority—if not sole—purchaser. This puts healthcare institutions in a position of significant power *as consumers*; it is not just the case that healthcare resource use is shaped by activity in other sectors; health care is large enough that its demands and practices can shape those sectors. If health institutions prioritise community‐owned, renewable energy supply, it will put suppliers who can meet those demands at a significant advantage. Providing seasonal, plant‐based food will create demand for those working to build more sustainable food systems. Hospitals often occupy large tracts of land within major urban areas and serve as transport hubs for those areas; this allows them to act as anchor institutions reshaping those spaces and transport networks.^76^

## 5 CONCLUSION

In describing the ‘bilocated birth’ of bioethics,^77^ Van Rensellaer Potter sketched two contrasting visions of what bioethics could and should achieve.^78^ The dominant model, focussing on immediate dilemmas arising in clinical practice, healthcare research, and policy, assumes that healthcare decisionmaking can occur in relative isolation from social and environmental processes, against a fixed background context.

Our entry into the Anthropocene demonstrates the violation of that assumption and justifies Potter's assertion that failure to adjust our perception of bioethics will result in a condition of ‘miserable survival’ (if, indeed, we survive at all). In the above, I have argued (as would Potter) that Aldo Leopold's Land Ethic provides some of the tools needed to shift our perceptions of the moral landscape in a way to meet the ethical challenges of this epoch. A diachronic, holistic, and biocentric ethic that positions us as members of a planetary ecological community encourages us to rethink our structures of health and care—and to resist holding the two as synonymous. The planetary boundaries framework provides the means to operationalise and quantify what is required from us to maintain the ‘integrity, stability, and beauty’ of the biotic communities to which we all belong.

## CONFLICT OF INTEREST STATEMENT

A. W. is a member of health workers' campaigning organisation Medact.

## Footnotes

1 Leopold, A. (1949). *A sand county almanac and sketches here and there*. Oxford University Press.

2 Bonneuil, C., & Fressoz, J.‐B. (2017). *The shock of the Anthropocene: The earth, history and us* (Reprint edition). Verso.

3 Waters, C. N., Zalasiewicz, J., Summerhayes, C., Barnosky, A. D., Poirier, C., Gałuszka, A., Cearreta, A., Edgeworth, M., Ellis, E. C., Ellis, M., Jeandel, C., Leinfelder, R., McNeill, J. R., Richter, D. d., Steffen, W., Syvitski, J., Vidas, D., Wagreich, M., Williams, M., … Wolfe, A. P. (2016). The Anthropocene is functionally and stratigraphically distinct from the Holocene. *Science*, *351*(6269), aad2622. https://doi.org/10.1126/science.aad2622

4 Ibid.

5 Stengers, I. (2015). *In catastrophic times: Resisting the coming barbarism* (A. Goffey, Trans.). Open Humanities Press. http://dx.medra.org/10.14619/016. ‘Gaia’ here—named after the personification of Earth in Greek mythology—refers both generally to the idea of Earth as agent and not simply resource in our moral reasoning, but also to the Gaia of James Lovelock's hypothesis, in which the planetary ecosystem is understood as a single self‐regulating complex system, of which both organisms and inorganic constituents form interacting parts.

6 Wardrope, A. (2020). Health justice in the Anthropocene: Medical ethics and the land ethic. *Journal of Medical Ethics*, *46*(12), 791–796. https://doi.org/10.1136/medethics-2020-106855; Gardiner, S. M. (2011). *A perfect moral storm: The ethical tragedy of climate change*. Oxford University Press.

7 Munthe, C., Fumagalli, D., & Malmqvist, E. (2021). Sustainability principle for the ethics of healthcare resource allocation. *Journal of Medical Ethics*, *47*(2), 90–97. https://doi.org/10.1136/medethics-2020-106644; Resnik, D. B. (2012). *Environmental health ethics*. Cambridge University Press.

8 Bonneuil & Fressoz, op. cit. note 2, p. 288.

9 Leopold, op. cit. note 1.

10 Kukla, R. (2005). Conscientious autonomy: Displacing decisions in health care. *Hastings Center Report*, *35*(2), 34–44. https://doi.org/10.1353/hcr.2005.0025

11 Callicott, J. B. (1989). The conceptual foundations of the Land Ethic. In *In defense of the land ethic* (pp. 75–100). State University of New York Press.

12 Wardrope, A. (2019). Does clinical ethics need a Land Ethic? *Medicine, Health Care and Philosophy*, *22*(4), 531–543. https://doi.org/10.1007/s11019-019-09890-x

13 A set of interrelated earth systems processes that jointly define the conditions within which ecosystems remain resilient and capable of supporting human civilisation.

14 Rockström, J., Steffen, W., Noone, K., Persson, Å., Chapin, F. S., Lambin, E. F., Lenton, T. M., Scheffer, M., Folke, C., Schellnhuber, H. J., Nykvist, B., de Wit, C. A., Hughes, T., van der Leeuw, S., Rodhe, H., Sörlin, S., Snyder, P. K., Costanza, R., Svedin, U., … Foley, J.A. (2009). A safe operating space for humanity. *Nature*, *461*(7263), 472–475. https://doi.org/10.1038/461472a

15 Gardiner, op. cit. note 6.

16 I address this in more detail elsewhere. Wardrope, A. (2022). Medical ethics and the land ethic. In D. C. Poff & A. C. Michalos (Eds.), *Encyclopedia of business and professional ethics* (pp. 1–6). Springer International Publishing. https://doi.org/10.1007/978-3-319-23514-1_1317-1; Wardrope, op. cit. note 6; Wardrope, op. cit. note 12.

17 Wardrope, op. cit. note 16.

18 The features I suggest ‘modern bioethics’ must challenge interestingly mirror closely those of ‘modern epidemiology’ challenged by Tony McMichael, one of the pioneers of ‘planetary health’, at the turn of the century. I am grateful to an anonymous reviewer for this point. (See: McMichael, A. J. (1999). Prisoners of the proximate: Loosening the constraints on epidemiology in an age of change. *American Journal of Epidemiology*, *149*(10), 887–897. https://doi.org/10.1093/oxfordjournals.aje.a009732).

19 Wardrope, op. cit. note 6; Wardrope, op. cit. note 12.

20 Warren, V. L. (n.d.). Feminist directions in medical ethics*. *Hypatia*, *4*(2), 73–86. https://doi.org/10.1111/j.1527-2001.1989.tb00574.x

21 Kukla, op. cit. note 10, p. 41.

22 The World Commission on Environment and Development. (1987). *Our common future*. Oxford University Press.

23 Wardrope, op. cit. note 6.

24 Leopold, A. (1991). *The river of the mother of God: And other essays by Aldo Leopold*. University of Wisconsin Press.

25 Singer, P. (1972). Famine, affluence, and morality. *Philosophy & Public Affairs*, *1*(3), 229–243.

26 Leopold, op. cit. note 1, p. 341.

27 Ibid: 204.

28 Beever, J., & Morar, N. (2016). Bioethics and the challenge of the ecological individual. *Environmental Philosophy*, *13*(2), 215–238. https://doi.org/10.5840/envirophil201692937; Beever, J., & Morar, N. (2016). The porosity of autonomy: Social and biological constitution of the patient in biomedicine. *The American Journal of Bioethics: AJOB*, *16*(2), 34–45. https://doi.org/10.1080/15265161.2015.1120793

29 Beauchamp, T. L., & Childress, J. F. (2019). *Principles of biomedical ethics* (8th ed.). Oxford University Press.

30 Leopold, op. cit. note 1, pp. 176–177. Leopold's approach here is discussed further in Callicott, J. B. (1989). Hume's is/ought dichotomy and the relation of ecology to Leopold's land ethic. In *In defense of the land ethic* (pp. 117–128). State University of New York Press.

31 Leopold, op. cit. note 1, p. 204.

32 I opt for the term ‘holism’ to describe this concept given the long tradition of its use by both proponents and detractors of the Land Ethic to describe its assignment of intrinsic value to communities, the essential dependence of individuals on the collective function of their mutual inderdependents, and the consequence rejection of individualism. The terminology echoes Leopold's own (e.g., in his essay ‘Conservation: In whole or in part?’ [(1991). *The river of the mother of God: And other essays by Aldo Leopold* (p. 310ff). University of Wisconsin Press], which outlines his theory of land health along the holistic lines sketched above.) However it is important to distinguish this from the metaphysical holism (and its political sequelae) of Jan Smuts. I am grateful to an anonymous reviewer for this point.

33 Stern, N. (2007). Economics, ethics and climate change. In *The economics of climate change: The stern review* (pp. 25–45). Cambridge University Press. https://doi.org/10.1017/CBO9780511817434.006

34 Parfit, D. (1984). *Reasons and persons*. Oxford University Press.

35 Callicott, J. B. (2014). *Thinking like a planet: The land ethic and the earth ethic*. Oxford University Press.

36 Callahan, D. (2003). Individual good and common good: A communitarian approach to bioethics. *Perspectives in Biology and Medicine*, *46*(4), 496–507. https://doi.org/10.1353/pbm.2003.0083

37 Richie, C. (2019). *Principles of green bioethics: Sustainability in health care*. Michigan State University Press. https://doi.org/10.14321/j.ctvhrd1wq; Resnik, op. cit. note 7.

38 Leopold, op. cit. note 24, p. 209.

39
*Pace* Ship of Theseus‐type arguments!

40 I am grateful to an anonymous reviewer for highlighting this point.

41 Callicott, J. B. (2014). The earth ethic: A critical account of its anthropocentric foundations—Responsibility to future generations and for global human civilization. In J. B. Callicott (Ed.), *Thinking like a planet: The land ethic and the earth ethic*. Oxford University Press. https://doi.org/10.1093/acprof:oso/9780199324880.003.0012

42 Leopold, op. cit. note 24, pp. 268–269.

43 Leopold, op. cit. note 1, pp. 24–25.

44 For example, Richie, op. cit. note 37; Resnik, op. cit. note 7; Dwyer, J. (2009). How to connect bioethics and environmental ethics: Health, sustainability, and justice. *Bioethics*, *23*(9), 497–502. https://doi.org/10.1111/j.1467-8519.2009.01759.x

45 Munthe, C., et al., op. cit. note 7.

46 Leopold, op. cit. note 1, p. 121.

47 Leopold discusses several such examples from his own work in his essay ‘The Ecological Conscience’; Leopold, op. cit. note 24.

48 Richardson, K., Steffen, W., Lucht, W., Bendtsen, J., Cornell, S. E., Donges, J. F., Drüke, M., Fetzer, I., Bala, G., von Bloh, W., Feulner, G., Fiedler, S., Gerten, D., Gleeson, T., Hofmann, M., Huiskamp, W., Kummu, M., Mohan, C., Nogués‐Bravo, D., … Rockström, J. (2023). Earth beyond six of nine planetary boundaries. *Science Advances*, *9*(37), eadh2458. https://doi.org/10.1126/sciadv.adh2458

49 Pattnaik, A., & Mulcahy, E. (2023). *Biodiversity, climate change, and health*. UK Health Alliance on Climate Change.

50 Cohen, A. J., Brauer, M., Burnett, R., Anderson, H.R., Frostad, J., Estep, K., Balakrishnan K., Brunekreef, B., Dandona, L., Dandona, R., Feigin, V., Freedman, G., Hubbell, B., Jobling, A., Kan, H., Knibbs, L., Liu, Y., Martin, R., Morawska, L., … Forouzanfar, M. H. (2017). Estimates and 25‐year trends of the global burden of disease attributable to ambient air pollution: An analysis of data from the Global Burden of Diseases Study 2015. *Lancet*, *389*(10082), 1907–1918. https://doi.org/10.1016/S0140-6736(17)30505-6

51 Romanello, M., Napoli, C. D., Drummond, P., Green, C., Kennard, H., Lampard, P., Scamman, D., Arnell, N., Ayeb‐Karlsson, S., Ford, L. B., Belesova, K., Bowen, K., Cai, W., Callaghan, M., Campbell‐Lendrum, D., Chambers, J., van Daalen, K. R., Dalin, C., Dasandi, N., … Costello, A. (2022). The 2022 report of the Lancet Countdown on health and climate change: health at the mercy of fossil fuels. *The Lancet*, *400*(10363), 1619–1654. https://doi.org/10.1016/S0140-6736(22)01540-9

52 Lade, S. J., Steffen, W., de Vries, W., Carpenter, S. R., Donges, J. F., Gerten, D., Hoff, H., Newbold, T., Richardson, K., & Rockström, J. (2020). Human impacts on planetary boundaries amplified by Earth system interactions. *Nature Sustainability*, *3*(2), 119–128. https://doi.org/10.1038/s41893-019-0454-4

53 Lade, S. J., Norberg, J., Anderies, J. M., Beer, C., Cornell, S. E., Donges, J. F., Fetzer, I., Gasser, T., Richardson, K., Rockström, J., & Steffen, W. (2019). Potential feedbacks between loss of biosphere integrity and climate change. *Global Sustainability*, *2*, e21. https://doi.org/10.1017/sus.2019.18; Anderies, J. M., Carpenter, S. R., Steffen, W., & Rockström, J. (2013). The topology of non‐linear global carbon dynamics: from tipping points to planetary boundaries. *Environmental Research Letters*, *8*(4), 044048. https://doi.org/10.1088/1748-9326/8/4/044048

54 Leopold, op. cit. note 1.

55 Lenzen, M., Malik, A., Li, M., Fry, J., Weisz, H., Pichler, P.‐P., Chaves, L. S. M., Capon, A., & Pencheon, D. (2020). The environmental footprint of health care: A global assessment. *The Lancet Planetary Health*, *4*(7), e271–e279. https://doi.org/10.1016/S2542-5196(20)30121-2

56 Dzau, V. J., Levine, R., Barrett, G., & Witty, A. (2021). Decarbonizing the U.S. Health Sector—A Call to Action. *New England Journal of Medicine, 85*(23), 2117–2119. https://doi.org/10.1056/NEJMp2115675

57 Lenzen, M., et al., op. cit. note 55.

58 IPCC. (2023). Summary for Policymakers. In Core writing team, H. Lee, & J. Romero (Eds.), *Climate Change 2023: Synthesis Report. Contribution of Working Groups I, II and II to the Sixth Assessment Report of the Intergovernmental Panel on Climate Change* (pp. 1–34). IPCC. https://doi.org/10.59327/IPCC/AR6-9789291691647.001

59 Wardrope, A., Mattam, T., Farid, M., Whitton, L., Harmer, A., Collins, F., Saleh, A., Abrams, R., & Aked, H. (2021). *Play the health card: The case for health and climate justice at the UN climate talks*. Medact. https://stat.medact.org/uploads/2021/10/Play-the-health-card-the-case-for-climate-health-justice-at-the-UN-Climate-Talks-MEDACT-H4GND-OCT2021.pdf

60 Richie, op. cit. note 37, p. 57.

61 Leopold, op. cit. note 1, p. viii.

62 Luta, X., Diernberger, K., Bowden, J., Droney, J., Howdon, D., Schmidlin, K., Rodwin, V., Hall, P., & Marti, J. (2024). Healthcare trajectories and costs in the last year of life: A retrospective primary care and hospital analysis. *BMJ Supportive & Palliative Care*, *14*, e807–e815. https://doi.org/10.1136/bmjspcare-2020-002630

63 Langton, J. M., Blanch, B., Drew, A. K., Haas, M., Ingham, J. M., & Pearson, S.‐A. (2014). Retrospective studies of end‐of‐life resource utilization and costs in cancer care using health administrative data: A systematic review. *Palliative Medicine*, *28*(10), 1167–1196. https://doi.org/10.1177/0269216314533813

64 Deptola, A. Z., & Riggs, J. (2019). Inpatient goals‐of‐care conversations reduce intensive care unit transfers in high‐risk patients. *American Journal of Hospice and Palliative Medicine®*, *36*(7), 583–586. https://doi.org/10.1177/1049909118824546

65 Jansen, J., Naganathan, V., Carter, S. M., McLachlan, A. J., Nickel, B., Irwig, L., Bonner, C., Doust, J., Colvin, J., Heaney, A., Turner, R., & McCaffery, K. (2016). Too much medicine in older people? Deprescribing through shared decision making. *BMJ*. *353*, i2893. https://doi.org/10.1136/bmj.i2893

66 Mortimer, F. (2010). The sustainable physician. *Clinical Medicine*, *10*(2), 110–111. https://doi.org/10.7861/clinmedicine.10-2-110

67 Wardrope, A. (2015). Autonomy as ideology: Towards an autonomy worthy of respect. *The New Bioethics*, *21*(1), 56–70. https://doi.org/10.1179/2050287715Z.00000000057

68 Potter, V.R. (1988). *Global bioethics: Building on the leopold legacy*. Michigan State University Press. Potter concentrates heavily on these ameliorative changes in his understanding of the Land Ethic, with a particular emphasis on fertility and contraception. Subsequent developments have shown that overpopulation poses less of an environmental threat than was assumed by many in Potter's era, hence my focus rather on specific anthropogenic influences and the earth systems they disrupt.

69 Parens, E. (2013). On good and bad forms of medicalization. *Bioethics*, *27*(1), 28–35. https://doi.org/10.1111/j.1467-8519.2011.01885.x

70 Wardrope, A. (2017). Mistaking the map for the territory: What society does with medicine. *International Journal of Health Policy and Management*, *6*(10), 605–607. https://doi.org/10.15171/ijhpm.2017.20

71 Kaczmarek, E. (2022). Promoting diseases to promote drugs: The role of the pharmaceutical industry in fostering good and bad medicalization. *British Journal of Clinical Pharmacology*, *88*(1), 34–39. https://doi.org/10.1111/bcp.14835; Moynihan, R., G⊘tzsche, P. C., Heath, I., & Henry, D. (2002). Selling sickness: The pharmaceutical industry and disease mongeringCommentary: Medicalisation of risk factors. *BMJ*, *324*(7342), 886–891. https://doi.org/10.1136/bmj.324.7342.886

72 Conrad, P. (1992). Medicalization and social control. *Annual Review of Sociology*, *18*(1), 209–232. https://doi.org/10.1146/annurev.so.18.080192.001233

73 Wardrope, A. (2015). Medicalization and epistemic injustice. *Medicine, Health Care and Philosophy*, *18*(3), 341–352. https://doi.org/10.1007/s11019-014-9608-3

74 Sinnott‐Armstrong, W. (2005). It's not my fault: Global warming and individual moral obligations. In W. Sinnott‐Armstrong & R. Howarth (Eds.), *Perspectives on climate change* (pp. 221–253). Elsevier.

75 Gunja, M., Gumas, E., & Williams, R. D, II. (2023, January 31). *U.S. health care from a global perspective, 2022: Accelerating spending, worsening outcomes*. https://doi.org/10.26099/8ejy-yc74

76 Abrams, R., Adhikari, R., Aked, H., Angharad, L., Barker, R., Collins, F., Eder, B., Elliott, T., Göpfert, A., Hadley, D., Harmer, A., O'Neill, E., Page, B., Saleh, A., Sharman, M., van Schalkwyk, M., Stanford, V., Wardrope, A., Whitaker, B., & Wood, E. (2021). *The public health case for a green new deal*. Medact. https://stat.medact.org/uploads/2021/04/The-public-health-case-for-a-Green-New-Deal-MEDACT-April-2021.pdf

77 Reich, W. T. (1995). The word ‘bioethics’: The struggle over its earliest meanings. *Kennedy Institute of Ethics Journal*, *5*(1), 19–34.

78 Potter, op. cit. note 70.

